# Design, Modeling, and Fabrication of High-Speed VCSEL with Data Rate up to 50 Gb/s

**DOI:** 10.1186/s11671-019-3107-7

**Published:** 2019-08-14

**Authors:** Chih-Chiang Shen, Tsung-Chi Hsu, Yen-Wei Yeh, Chieh-Yu Kang, Yun-Ting Lu, Hon-Way Lin, Hsien-Yao Tseng, Yu-Tzu Chen, Cheng-Yuan Chen, Chien-Chung Lin, Chao-Hsin Wu, Po-Tsung Lee, Yang Sheng, Ching-Hsueh Chiu, Hao-Chung Kuo

**Affiliations:** 1 Department of Photonics & Graduate Institute of Electro-Optical Engineering, College of Electrical and Computer Engineering, National Chiao Tung University, Hsinchu, 30010 Taiwan; 2 Institute of Photonic System, National Chiao Tung University, Tainan, 71150 Taiwan; 3 Graduate Institute of Photonics and Optoelectronics, National Taiwan University, Taipei, 10617 Taiwan; 4 Crosslight Software Inc., China Branch, Shanghai, 200063 China

**Keywords:** High-speed VCSEL, PICS3D, 50 Gb/s, Oxide aperture, Cavity length

## Abstract

We have studied the characteristics of frequency response at 850-nm GaAs high-speed vertical-cavity surface-emitting lasers (VCSELs) with different kinds of oxide aperture sizes and cavity length using the PICS3D simulation program. Using 5-μm oxide aperture sizes, the frequency response behavior can be improved from 18.4 GHz and 15.5 GHz to 21.2 GHz and 19 GHz in a maximum of 3 dB at 25 °C and 85 °C, respectively. Numerical simulation results also suggest that the frequency response performances improved from 21.2 GHz and 19 GHz to 30.5 GHz and 24.5 GHz in a maximum of 3 dB at 25 °C and 85 °C due to the reduction of cavity length from 3λ/2 to λ/2. Consequently, the high-speed VCSEL devices were fabricated on a modified structure and exhibited 50-Gb/s data rate at 85 °C.

## Introduction

In a few years, the vertical-cavity surface-emitting laser diodes (VCSELs) have become favorite transmitters for optical data links [1, 2]. Meanwhile, GaAs VCSEL devices have some advantages like low threshold current, power consumption, and small divergence angle, as well as top side illumination easily to make an array. Its demand has grown rapidly along with huge requirements for 5G Internet, 3D sensing, LiDAR, high-speed photodetectors, etc. [3–14].

PICS3D (Photonic Integrated Circuit Simulator in 3D) is a state-of-the-art 3D simulator for laser diodes and related active photonic devices. PISC3D is a 3D comprehensive numerical solver offering rigorous and self-consistent treatment on thermal, electrical, and optical properties by solving the related equations based on the nonlinear Newton-Raphson method. Its primary goal is to provide a 3D simulator for edge- and surface-emitting laser diodes. It has also been expanded to include models for other components integrated with or related to the laser emitter. In this study, we simulated GaAs VCSEL; of course, it also expanded easily to GaN VCSEL, LED, etc. [15, 16].

The first oxidation process in III–V compound material was discovered at the University of Illinois at Urbana-Champaign by Dallesasse and Holonyak in 1989 [17]. Through an oxidation process, the VCSEL devices can narrow down the size of oxide aperture diameter. Thus, it can not only promote a single transverse mode operation but also high-speed operation and single-mode performance.

To achieve a high modulation bandwidth, most designers would seek a large D-factor and reasonable low K-factor, typically a high differential gain by using strain QWs. A low photon lifetime by tuning the phase of the top distributed Bragg reflector (DBR) [18], a high confinement factor by employing a short cavity, and a small cavity oxide are necessary. On the other hand, reducing electrical parasitics can also improve modulation speed. These include parasitic capacitance from bond pads, intrinsic diode junction, and the area of out of aperture below metal contact pads which connects DBRs, oxidation layers, etc., and also include parasitic resistance from DBRs, junction resistance. However, parasitic resistance is not better as low as possible; it needs to match 50 Ohm impedance. Regarding the high-speed VCSEL device development for data communication, there are several papers that record the progress [19, 20]. Today, the state-of-the-art 50-Gb/s 850-nm VCSEL devices have been demonstrated successfully at Chalmers University of Technology (CUT) by Westbergh et al. and University of Illinois Urbana-Champaign (UIUC) by Feng et al. [21–23]. We compared our experiments’ result in this study with other labs, and our data is much close to their results.

However, the most effective way to increase the differential gain is the use of strain multiple quantum well (MQW), such as replacing the GaAs/AlGaAs MQW by the InGaAs/AlGaAs MQW [24, 25]. In the GaAs-based material, the hole effective mass is much larger than the electron effective mass, which causes the quasi-Fermi level to separate toward the valance band [26]. Hence, if we implement the strain on an active layer, the effective hole mass can be reduced significantly because the separation of the quasi-Fermi level is more balanced between the conduction and valance band. The differential gain can be considered as the growth of gain with carrier density once the quasi-Fermi level separation becomes more symmetric, and in the meanwhile, the differential gain will become more compressive in the strained MQW. Furthermore, the strain will also release the valance band mixing effect by increasing the energy difference between the heavy hole and light hole band. In this study, the numerical simulation was optimized to the VCSEL device structure through Crosslight PICS3D software [27].

## Methods/experimental

**Fig. 1:**
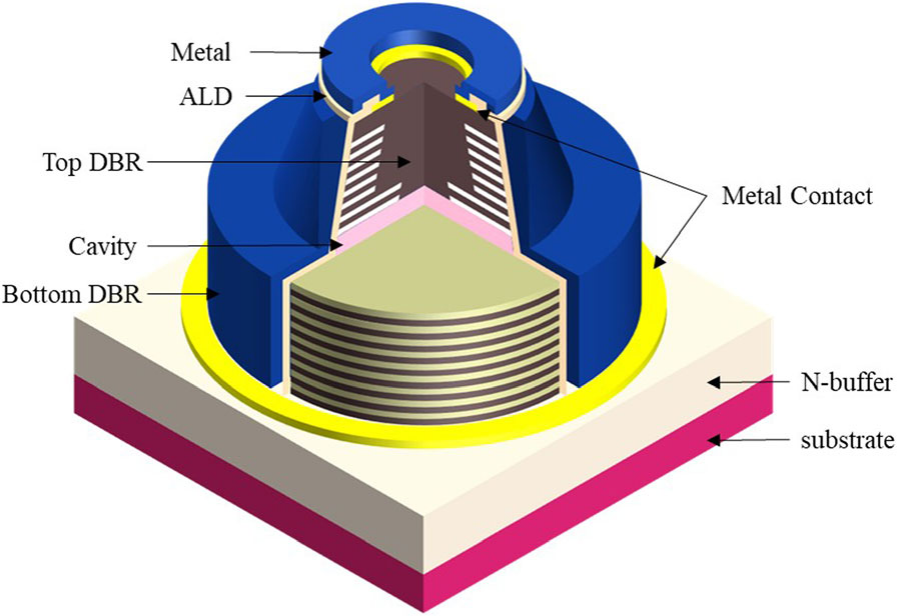
The schematic of the top emitting 850-nm VCSEL

Figure 1 shows the schematic of the 850-nm GaAs VCSEL device for simulation structure in this study. For this oxide VCSEL, the epitaxial layer structure from bottom to top includes a GaAs substrate, n-DBR of 34 pairs of Al_0.9_Ga_0.1_As/Al_0.12_Ga_0.88_As, an InGaAs MQW active layer with five In_0.08_Ga_0.92_As-strained QWs separated by six Al_0.37_Ga_0.63_As quantum barrier layers, p-DBR, and a heavily doped p-GaAs as a contact layer. However, p-DBR layers include two Al_0.98_Ga_0.02_As oxidation layers and four Al_0.96_Ga_0.04_As oxidation layers and 13 pairs of Al_0.9_Ga_0.1_As/Al_0.12_Ga_0.88_As layers. There are two kinds of oxide aperture sizes, 5 μm and 7 μm in our design. The two Al_0.98_Ga_0.02_As oxidation layers would get an aperture confinement for the functions of electrical and optical, and the four Al_0.96_Ga_0.04_As layers would reduce parasitic capacitance and further improve the optical response. Thus, we calculate the electrical potential and charge distribution via Poisson’s equation, calculate carrier transport from the current continuity equations, use effective index method (EIM) approximation which has been successfully applied to calculate various VCSEL structures, and utilize the transfer-matrix method in the calculation of equivalent laser cavity. In this study, applied to perform our VCSEL simulations was the VCSEL modules in Crosslight PICS3D software which includes quantum mechanical, electrical, thermal, and DBR cavity optical effects, with stronger self-consistent interaction than any other optoelectronic devices that were applied to perform our VCSEL simulations. Considering that the simulated VCSEL structure is symmetric, cylindrical coordinate system, instead of the Cartesian coordinate system, was used for the sake of saving simulation time. The sophisticated Newton iteration formula was utilized in the software to ensure the correct answers to be found in nonlinear equations in the VCSEL module. In this report, we have especially considered different kinds of oxide aperture sizes and cavity lengths for improving VCSEL device performance. The VCSEL A and B are designed for 7-μm and 5-μm oxide aperture with 3λ/2 cavity length, respectively. On the other hand, VCSEL C adopts the design of 5-μm oxide aperture with λ/2 cavity length.

## Results and Discussion

**Fig. 2:**
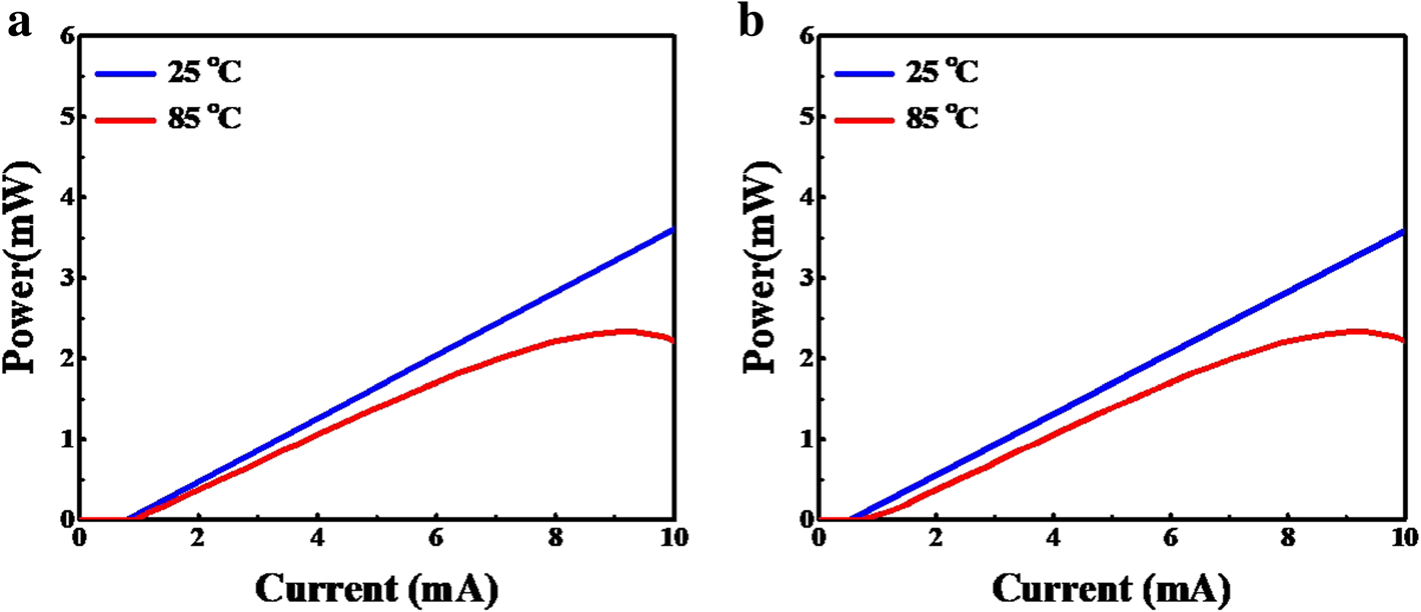
The light-current characteristics for the simulation of VCSEL devices with 3λ/2 cavity length for **a** VCSEL A: 7 μm aperture diameter at 25 °C and at 85 °C, and **b** VCSEL B: 5 μm aperture diameter at 25 °C and at 85 °C

**Fig. 3:**
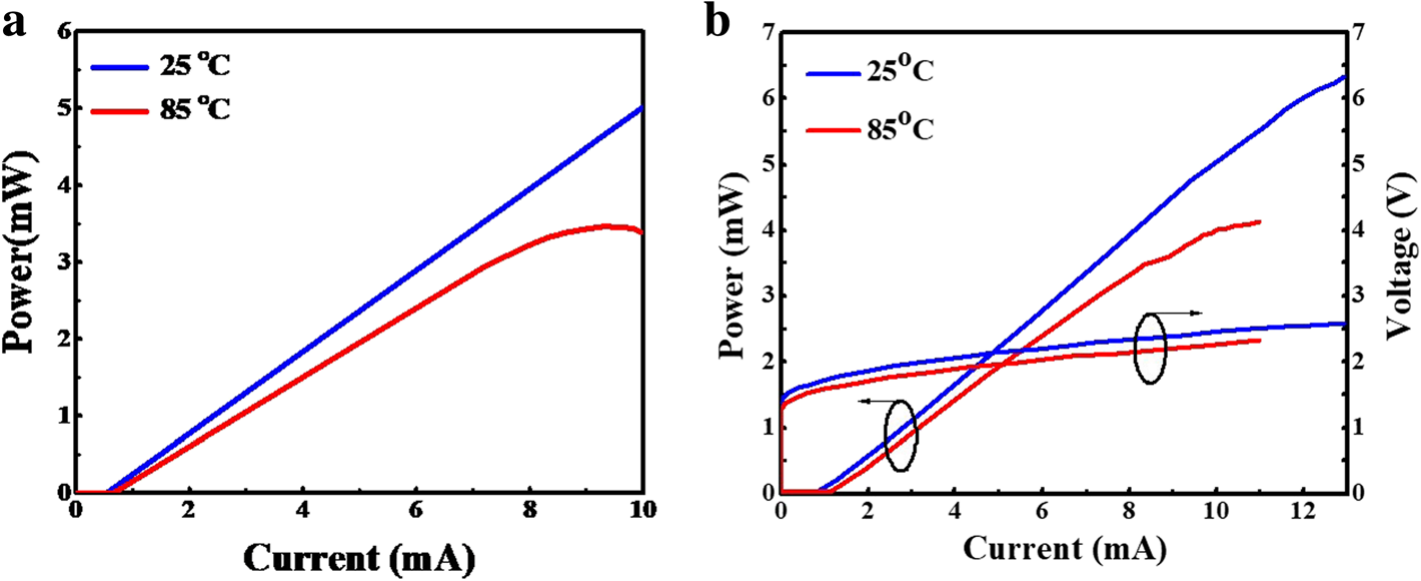
**a** The light-current characteristics for the simulation of VCSEL C: λ/2 cavity length, 5 μm aperture diameter at 25 °C and 85 °C. **b** The measured light-current-voltage characteristics of VCSEL C at 25 °C and 85 °C

In VCSEL A and B, their cavity lengths are 3λ/2 but have different oxide aperture diameters 7 μm (VCSEL A) and 5 μm (VCSEL B), respectively. From simulation results, L-I curves are depicted in Fig. 2 a and b. We can see the threshold current of VCSEL B (*I*_th_ 0.6 mA and 0.73 mA) is always lower than the VCSEL A (*I*_th_ 0.82 mA and 0.94 mA) at 25 °C and 85 °C, respectively. Obviously, the *I*_th_ becomes bigger along with increasing oxide aperture size. To achieve the smallest possible mode-volume in the vertical direction and increase the D-factor, a short λ/2 optically thick cavity is used and then fixed at the 5-μm oxide aperture in VCSEL C. From the L-I curve, we can see the threshold current of VCSEL C (*I*_th_ 0.55 mA and 0.67 mA) are always lower than the VCSEL B (*I*_th_ 0.6 mA and 0.73 mA) at 25 °C and 85 °C, respectively, as shown in Fig. 3 a. In the experiment data of VCSEL C (real), L-I-V curves are shown in Fig. 3 b, the *I*_th_ of VCSEL C (real) are 0.8 mA and 1.08 mA at 25 °C and 85 °C, respectively. In the real case, because the thermal effect may induce the difference of *I*_th_ between the real case and simulation, results can be expected.

According to resonance frequency (*fr*) and damping rate function,

1 $$ fr=D\bullet \sqrt{I-{I}_{\mathrm{th}}}\ \mathrm{where}\ D=\frac{1}{2\pi}\bullet \sqrt{\frac{\eta_i\Gamma {V}_g}{q{V}_a}\bullet \frac{\partial g}{\partial n}} $$

2 $$ \gamma =K\bullet {f_r}^2+{\gamma}_o\ \mathrm{where}\ K=4{\pi}^2\left({\tau}_p+\frac{\varepsilon }{v_g\left(\raisebox{1ex}{$\partial g$}\!\left/ \!\raisebox{-1ex}{$\partial n$}\right.\right)}\right) $$

where *D* is the D-factor, *I* is the current, *I*_th_ is the threshold current, *η*_*i*_ is the internal quantum efficiency, *Г* is the optical confinement factor, *V*_*g*_ is the group velocity, *q* is the elementary charge, *V*_*a*_ is the volume of the active (gain) region, $\frac{\partial g}{\partial n}$ is the differential gain, *γ* is the damping factor, *K* is the K-factor, *γ*_*o*_ is the damping factor offset, *τ*_*p*_ is the photon lifetime, and *ε* is the gain compression factor [28].

**Fig. 4:**
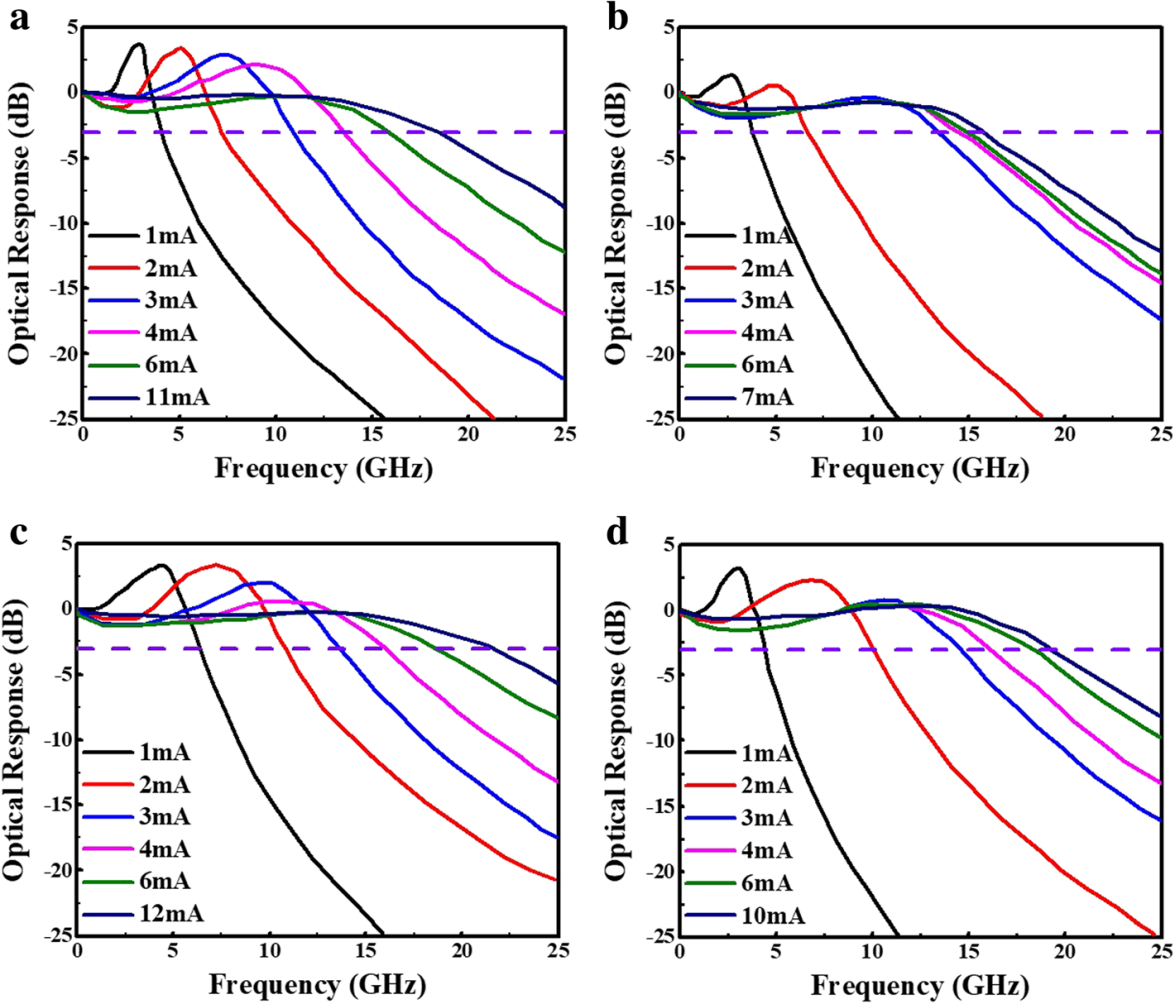
Simulation of small-signal modulation response for VCSEL devices with 3λ/2 cavity length; thus, VCSEL A and B are with 7 μm and 5 μm aperture diameter, respectively, for VCSEL A at **a** 25 °C and at **b** 85 °C, and for VCSEL B at **c** 25 °C and at **d** 85 °C.

Thus, we can improve the frequency response of device performances by reducing the lifetime of photon and the effective volume of the resonator and increasing differential gain. Based on these considerations, we use the same parameters for the next section to improve the optical response. Figure 4 a–d shows the small-signal modulation response of VCSEL A and VCSEL B at 25 °C and 85 °C. From the simulation result of high-speed optical response, it has a good 3-dB bandwidth from 18.4 GHz and 15.5 G Hz (VCSEL A) to 21.2 GHz and 19 GHz (VCSEL B) and it also indicates the 3-dB bandwidth was enhanced by approximately 15.2% and 22.5%, respectively. Thus, attributed to the increasing confinement factor, the VCSEL devices have the lower threshold current in the emission and the batter bandwidth in VCSEL can be attributed to the confinement factor increased using smaller oxide aperture size.

**Fig. 5:**
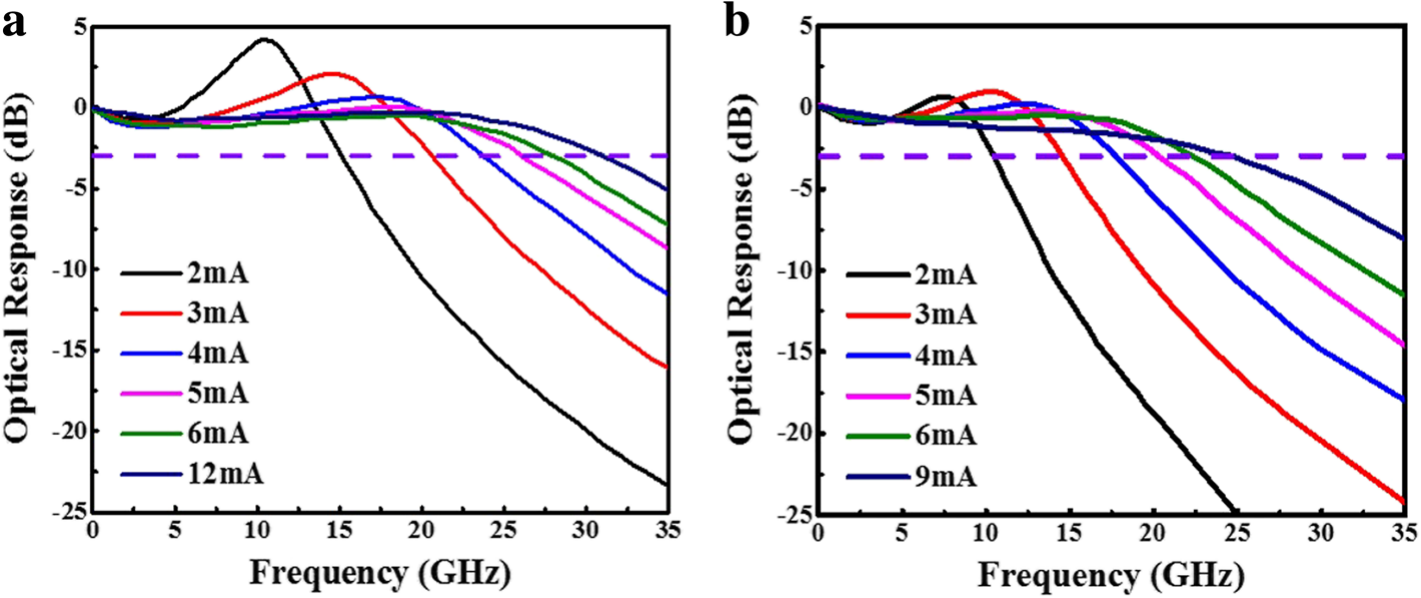
Simulation of small-signal modulation response for VCSEL C: λ/2 cavity length, 5 μm aperture diameter at **a** 25 °C and at **b** 85 °C

In the following case, we keep the 5-μm oxide aperture and shorten the cavity length to λ/2. Figure 5 a and b shows the small-signal modulation response of VCSEL C at 25 °C and 85 °C. From the simulation result of high-speed optical response, it has a good 3-dB bandwidth from 21.2 GHz and 19 GHz (VCSEL B) to 30.5 GHz and 24.5 GHz (VCSEL C) and it also indicates the 3-dB bandwidth was enhanced by approximately 43.9% and 28.9%, respectively. Thus, both simulation results show that the VCSEL devices which have the lower threshold current and larger bandwidth attributed to the increasing confinement factor using shorter cavity length.

**Fig. 6:**
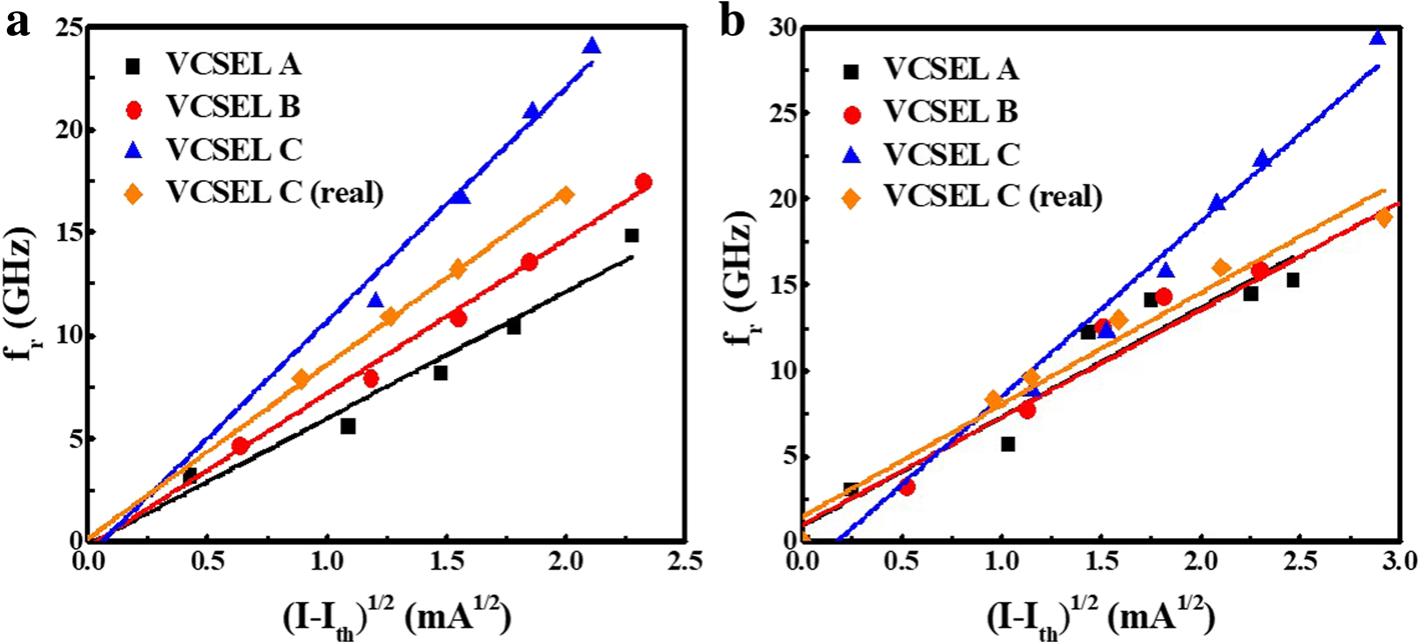
The 3-dB frequency versus the square root of (*I*-*I*_th_) of the simulation for VCSEL A, VCSEL B, VCSEL C, VCSEL C (real) at **a** 25 °C and **b** 85 °C

Figure 6 shows simulated f3dB versus the square root of (*I* − *I*_th_). The slope of these data points can be expressed as

3 $$ {\mathrm{f}}_{3\mathrm{dB}}=D\times \sqrt{I-{I}_{\mathrm{th}}} $$

The D-factor is an important parameter which related to internal quantum efficiency and the differential gain of the quantum wells for VCSEL operating at high speed [29]. Thus, the D-factor was 6.9, 7.3, and 11 GHz/mA^1/2^ at 25 °C for VCSEL A, B, and C devices, respectively. On the other hand, the D-factor was 6.0, 6.7, and 9.4 GHz/mA^1/2^ at 85 °C for VCSEL A, B, and C devices, respectively. From our results, the D-factor is inversely proportional to the oxide aperture diameter and cavity length. And the larger D-factor will be along with smaller threshold current. Furthermore, the VCSELs with smaller oxide aperture diameters (5 μm) and shorter cavity length (λ/2) are especially well-suited for data transmission at low energy per bit [30–32]. We expect the VCSEL can achieve error-free operation rate up to 50 Gb/s.

**Fig. 7:**
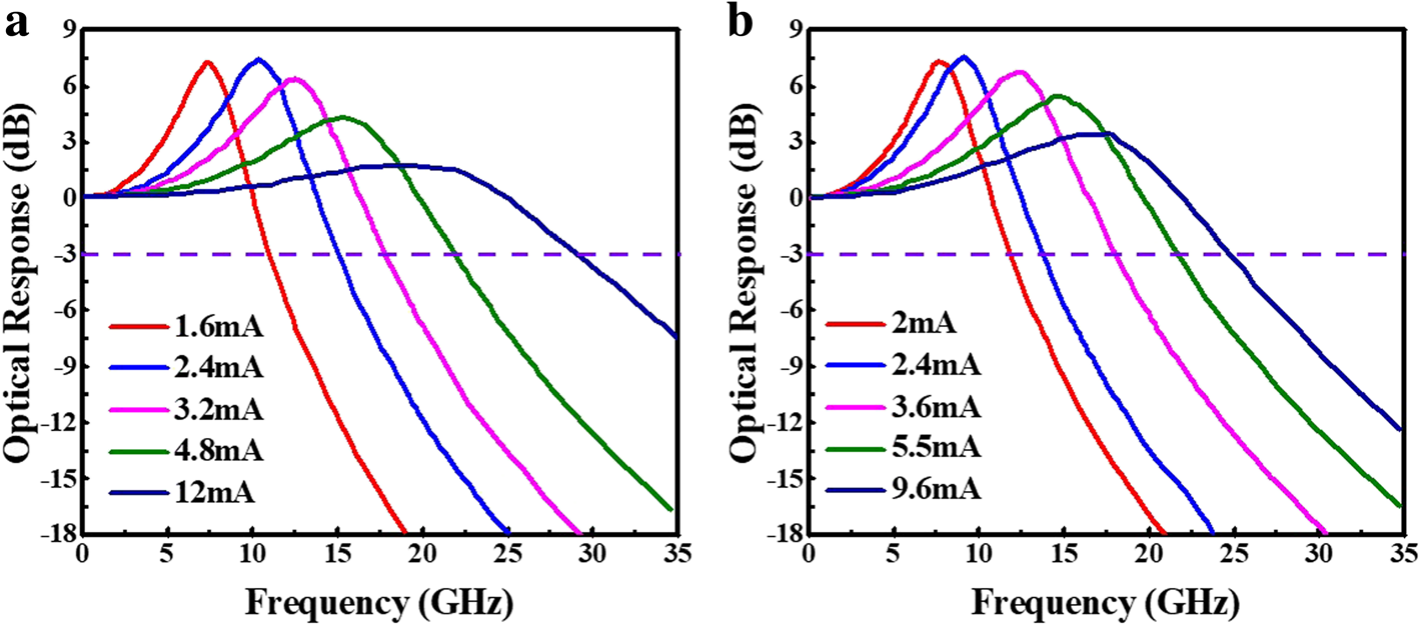
Measured small-signal modulation response for VCSEL C (real): λ/2 cavity length, 5 μm aperture diameter at **a** 25 °C and **b** 85 °C

Next, we fabricated the VCSEL device and compared the simulation result and real test data; next, we fabricated the VCSEL device. In Fig. 6, the D-factor of VCSEL C (real) was 8.5 and 8.3 GHz/mA^1/2^ at 25 °C and 85 °C, respectively. Figure 7 shows the measured small-signal modulation response at 25 °C and 85 °C. As we can see, the 3-dB bandwidth of measurement is 29.3 and 24.6 GHz at 25 °C and 85 °C, respectively. In the real device case, it was a little bit lower than the simulation case VCSEL C. The difference may come from the thermal effect and parasitic limitation due to device fabrication, as we mentioned earlier. Compared with others’ results, our simulation is closer to our own experiments [21–23]. This points out that our VCSEL simulation result can be applied for the high-speed laser.

## Conclusions

In conclusion, we optimized the oxide aperture and cavity length of the VCSEL structure by the PICS3D simulation program. Referring to these results, we fabricated 50-Gb/s VCSEL devices. The results showed a decrease in threshold current and improvement of 3-dB bandwidth in VCSEL devices. Finally, the high-speed VCSEL devices (up to 50-Gb/s data rate at 85 °C) had been demonstrated and successfully to create PICS3D model for 50-Gb/s VCSEL device design.

## Data Availability

In the current work, the data and analysis are available from the corresponding authors on reasonable request.

## Abbreviations

EIM: Effective index method
MQW: Multiple quantum well
PICS3D: Photonic Integrated Circuit Simulator in 3D
QWs: Quantum wells DBR Distributed Bragg reflector
VCSELs: Vertical-cavity surface-emitting lasers
